# Developing a gateway program for importing non‐animal origin ingredients from regions with African swine fever virus

**DOI:** 10.1111/tbed.14473

**Published:** 2022-02-23

**Authors:** Olivia L. Harrison, Jordan T. Gebhardt, Chad B. Paulk, Jason C. Woodworth, Cassandra K. Jones

**Affiliations:** ^1^ Department of Animal Sciences and Industry College of Agriculture Kansas State University Manhattan Kansas; ^2^ Department of Diagnostic Medicine/Pathobiology College of Veterinary Medicine Kansas State University Manhattan Kansas; ^3^ Department of Grain Science and Industry College of Agriculture Kansas State University Manhattan Kansas

**Keywords:** African swine fever virus, grains, import, oilseeds

## Abstract

The US Department of Agriculture (USDA) categorizes the risk of African swine fever virus (ASFV) entry into the United States through non‐animal origin feed ingredients as ‘negligible to moderate, with high uncertainty’. Both Canada and Australia have implemented policies that are suggested to reduce the risk of ASFV entry through feed ingredients, but the United States has not because of scientific limitations that have been addressed by recent publications. As regulators and industry consider a potential pathway forward, the objective of this manuscript is to describe a process to determine if a voluntary or regulatory import policy is warranted by the United States. Initially, the volume and types of non‐animal origin feed ingredients imported from countries with ASFV were quantified and assigned a level of risk (high risk: unprocessed grains and oilseeds, moderate risk: soybean co‐products (meals, oil, and oilcake), and low risk: amino acids, vitamins, and other synthetically produced products from countries that have ASFV). In 2020, moderate‐ and high‐risk ingredients from ASFV‐positive countries represented 3.1% of all ingredients imported into the United States. Policies from Canada and Australia were evaluated for practicality of implementation by US government officials. Industry representatives from both countries consistently stated their policies would not be feasible in the United States due to the differences in cost and complexity of the swine and feed industries. Overall, unprocessed, or high‐risk, ingredients from ASFV‐positive countries represent a low percentage of imported ingredients into the United States; however, cautionary procedures may still be warranted given industry demand.

## 1 INTRODUCTION

Currently, African Swine Fever virus (ASFV) has been confirmed in pigs in more than 50 countries. As the number of affected countries grows, so does the risk for virus entry into the United States. The most likely method of ASFV transmission is direct contact between pigs. In countries free from disease, prevention of entry is focused on preventing fomite‐based transmission. One potential route for fomite‐based transmission is the feed supply chain. The USDA cites that used tote bags carrying feed ingredients was the most likely source of porcine epidemic diarrhoea virus (PEDV) entry into the United States from China in 2013 (USDA‐APHIS, 2015). The virus was then spread throughout North America and remains endemic throughout the continent today. Research has shown that feed ingredients have the capability to sustain not only PEDV, but a host of other viruses, including ASFV (Dee et al., 2018).

In order for a feed ingredient to serve as a fomite in disease transmission, it must first be contaminated by the pathogen, that pathogen must survive transport to the point of consumption by an animal, and the quantity of virus consumed by an animal must be substantial enough to cause disease. These factors can vary widely in different types of ingredients. For example, wild boar or feral pigs affected by ASFV often seek food and shelter in fields. Their resulting carcasses may contaminate grains or seeds during harvest. Due to these differences, unprocessed grains or seeds from ASFV‐endemic regions are considered to be at a greater risk to be contaminated with the pathogen than manufactured ingredients, such as synthetic amino acids or vitamins, derived from the same region (EFSA, 2021). Virus survival also differs based on ingredient type. For example, the half‐life of ASFV can range from 9.6 to 12.9 days in conventional versus organic soybean meal (Stoian et al., 2019). Research has demonstrated that if ingredients are contaminated with a high level of ASFV, the virus can survive theoretical trans‐oceanic shipment to the United States (Dee et al., 2018). Finally, feed contaminated with ASFV has been demonstrated to cause infection in pigs when sufficient quantities of virus are ingested (Niederwerder et al., 2019).

These studies have raised concern that the feed supply chain may be a realistic route of ASFV entry into the United States. Both Canada and Australia have regulations to reduce this risk. Scientific limitations have slowed the implementation of similar rules in the United States. For example, there are no approved sampling or extraction protocols for ASFV detection in ingredients. Recent publications have helped to address these issues, and while some challenges still remain, there is greater confidence that a representative sample can be collected and analysed for ASFV if testing were allowed (Dee et al., 2021; Jones et al., 2019). This scientific progress led to the US Animal Health Association passing a resolution in 2020 that requested the federal government to ‘restrict the import of feed and/or a feed ingredient from countries that are positive for African swine fever and to create enforceable standards for those countries to reduce the contamination threat during harvest and processing of the feed and feed ingredients’. However, the government responded that there is too great of cost to both government and industry at the current time to implement such a program. Furthermore, the types of enforceable standards requested were too broad; there are mixed messages from within both the swine and feed industries about the types of standards that should be implemented. As regulators and industry consider a potential pathway forward, the objective of this manuscript is to describe a process to determine if a voluntary or regulatory import policy is warranted by the United States.

## 2 MATERIALS AND METHODS

To make the determination if an industrywide policy should be adopted, and what it should address, it was important to first quantify the sources and volumes of non‐animal origin ingredients imported into the United States from ASFV‐positive regions. Next, an assessment of the regulatory framework incorporated by other ASFV‐free countries was considered. Finally, a working group representing the swine industry made recommendations for a pilot import program and its eventual incorporation into regulatory policy.

### 2.1 The volume and path of non‐animal origin feed and ingredients imported from countries with ASFV

Import data from 2016 through 2020 were collected from the US International Trade Commission (USITC DataWeb) with the Harmonized Tariff Schedule (HTS) found in Table 1. Disease presence was based off OIE‐World Organization for Animal Health (OIE‐WAHIS) disease reporting database. Imported ingredients were separated into three categories based off their perceived risk of contamination or re‐contamination upon importation into the United States (Table 2). ‘Low‐risk’ ingredients were those that were least likely to be contaminated with ASFV, such as ingredients that are synthetically manufactured (amino acids, vitamins, enzymes) or undergo substantial thermal or chemical processing that would likely render pathogen inactive (oils and most oilseed cakes or flours). ‘Moderate‐risk’ ingredients were those that undergo thermal or chemical processing, but allow for the risk of post‐processing cross‐contamination and demonstrate a capacity for significant viral survival over time (soybean oilcake and soybean meal). ‘High‐risk’ ingredients were those that have a risk for natural contamination by affected animals and undergo minimal processing (whole grains or seeds). Notably, an exception was made to also characterize choline as a ‘high‐risk’ ingredient. Similar to vitamins, the synthetic production of choline poses little risk for ASFV contamination. While most vitamins are transported to the United States in their highly concentrated form and diluted upon arrival to the United States, choline is typically applied to a corncob or rice hull carrier shortly after manufacturing because it is highly hygroscopic. This application maximizes its stability during transoceanic shipment. The carrier to which choline applies has a similar probability of contamination as whole grains or seeds, so the imported product category of choline was included in the ‘high‐risk’ category.

**TABLE 1:** HTS codes utilized and their product descriptions

HTS code	Product description
1001.19	DURUM WHEAT, OTHER THAN SEED
1001.99	WHEAT AND MESLIN, NOT DURUM WHEAT, OTHER THAN SEED
1002.9	RYE, OTHER THAN SEED
1003.9	BARLEY, OTHER THAN SEED
1004.9	OATS, OTHER THAN SEED
1005.9	CORN (MAIZE), OTHER THAN SEED CORN
1006.1	RICE IN THE HUSK (PADDY OR ROUGH)
1007.9	GRAIN SORGHUM, OTHER THAN SEED
1008.1	BUCKWHEAT
1008.6	TRITICALE
1103	CEREAL GROATS, MEAL AND PELLETS
1104	CEREAL GRAINS, OTHERWISE WORKED (HULLED, ROLLED ETC.), EXCEPT RICE (HEADING 1006); GERM OF CEREALS, WHOLE, ROLLED, FLAKED OR GROUND
1107	MALT, WHETHER OR NOT ROASTED
1109	WHEAT GLUTEN, WHETHER OR NOT DRIED
1208	FLOURS AND MEALS OF OIL SEEDS OR OLEAGINOUS FRUITS, OTHER THAN THOSE OF MUSTARD
1201.90.0005	SOYBEAN SEEDS OF A KIND USED AS OIL STOCK, WHETHER OR NOT BROKEN
1201.90.0010	SOYBEANS, CERTIFIED ORGANIC, WHETHER OR NOT BROKEN, EXCEPT SEEDS OF A KIND USED FOR SOWING OR USED AS OIL STOCK
1201.90.0090	SOYBEANS, WHETHER OR NOT BROKEN, OTHER THAN CERTIFIED ORGANIC, NESOI
1204.00.0025	CERTIFIED ORGANIC FLAXSEED (LINSEED) FOR USE AS OIL STOCK, WHETHER OR NOT BROKEN
1204.00.0035	FLAXSEED (LINSEED) FOR USE AS OIL STOCK, WHETHER OR NOT BROKEN
1205.10.0020	RAPE OR COLZA SEEDS FOR USE AS OIL STOCK, LOW ERUCIC ACID, WHETHER OR NOT BROKEN
1205.90.0020	RAPE OR COLZA SEEDS FOR USE AS OIL STOCK, NESOI, WHETHER OR NOT BROKEN
1206.00.0020	SUNFLOWER SEEDS, WHETHER OR NOT BROKEN, FOR USE AS OIL STOCK
1207.60.0000	SAFFLOWER (CARTHAMUS TINCTORIUS) SEEDS, WHETHER OR NOT BROKEN
1507.10.0000	SOYBEAN OIL AND ITS FRACTIONS, CRUDE, WHETHER OR NOT DEGUMMED
1507.90.4020	SOYBEAN OIL AND ITS FRACTIONS, ONCE‐REFINED (SUBJECT TO ALKALAI OR CAUSTIC WASH BUT NOT BLEACHED OR DEODORIZED), NOT CHEMICALLY MODIFIED
1507.90.4040	SOYBEAN OIL AND ITS FRACTIONS, FULLY REFINED, WASHED, BLEACHED OR DEODORIZED BUT NOT CHEMICALLY MODIFIED, NESOI
2302.50.0000	BRAN, SHARPS (MIDDLINGS) AND OTHER RESIDUES, WHETHER OR NOT IN PELLETS, DERIVED FROM SIFTING, MILLING OR OTHER WORKINGS OF LEGUMINOUS PLANTS
2303.10.0010	CORN GLUTEN FEED, WHETHER OR NOT IN PELLETS
2303.10.0020	CORN GLUTEN MEAL, WHETHER OR NOT IN PELLETS
2304.00.0000	SOYBEAN OILCAKE AND OTHER SOLID RESIDUES RESULTING FROM THE EXTRACTION OF SOY BEAN OIL, WHETHER OR NOT GROUND OR IN THE FORM OF PELLETS
2306.10.0000	COTTON SEED OILCAKE AND OTHER SOLID RESIDUES RESULTING FROM THE EXTRACTION OF COTTON SEED OIL, WHETHER OR NOT GROUND OR IN THE FORM OF PELLETS
2306.20.0000	LINSEED OILCAKE AND OTHER SOLID RESIDUES RESULTING FROM THE EXTRACTION OF LINSEED OIL, WHETHER OR NOT GROUND OR IN THE FORM OF PELLETS
2306.30.0000	SUNFLOWER SEED OILCAKE AND OTHER SOLID RESIDUES RESULTING FROM THE EXTRACTION OF SUNFLOWER SEED OIL, WHETHER OR NOT GROUND OR IN THE FORM OF PELLETS
2306.41.0000	RAPE OR COLZA SEED OILCAKE AND OTHER SOLID RESIDUES, LOW ERUCIC ACID, RESULTING FROM THE EXTRACTION OF RAPE OR COLZA SEED OIL, WHETHER OR NOT GROUND
2306.49.0000	RAPE OR COLZA SEED OILCAKE AND OTHER SOLID RESIDUES, NESOI, RESULTING FROM THE EXTRACTION OF RAPE OR COLZA SEED OIL, WHETHER OR NOT GROUND/IN PELLETS
2306.90.0120	CORN (MAIZE) GERM OILCAKE AND OTHER SOLID RESIDUES RESULTING FROM THE EXTRACTION OF CORN OIL, WHETHER OR NOT GROUND OR IN THE FORM OF PELLETS
2309.90.7000	PREPARATIONS, WITH A BASIS OF VITAMIN B12, FOR SUPPLEMENTING ANIMAL FEED
2922.41.0010	LYSINE AND ITS ESTERS; SALTS THEREOF, MEETING REQUIREMENTS OF FOOD CHEMICAL CODEX, CODEX ALIMENTARIUS OR UNITED STATES PHARMACOPEIA
2922.41.0090	LYSINE AND ITS ESTERS; SALTS THEREOF, NESOI
2922.49.4950	AMINO ACIDS, NON‐AROMATIC, NESOI
2923.10.0000	CHOLINE AND ITS SALTS
2930.40.0000	METHIONINE
2933.99.1200	AROMATIC OR MODIFIED AROMATIC HETEROCYCLIC COMPOUNDS WITH NITROGEN HETERO ATOM(S) ONLY, EXCEPT ACRIDINE, INDOLE AND CARBAZOLE
2936.21.0000	VITAMINS A AND THEIR DERIVATIVES UNMIXED
2936.22.0000	VITAMIN B1 (THIAMINE) AND ITS DERIVATIVES
2936.23.0000	VITAMIN B2 (RIBOFLAVIN) AND ITS DERIVATIVES
2936.24.0000	D‐ OR DL‐PANTOTHENIC ACID (VITAMIN B3 OR VITAMIN B5) AND ITS DERIVATIVES
2936.25.0000	VITAMIN B6 (PYRIDOXINE AND RELATED COMPOUNDS WITH VITAMIN B6 ACTIVITY) AND ITS DERIVATIVES
2936.26.0000	VITAMIN B12 (CYANOCOBALAMIN AND RELATED COMPOUNDS WITH VITAMIN B12 ACTIVITY) AND ITS DERIVATIVES
2936.27.0000	VITAMIN C (ASCORBIC ACID) AND ITS DERIVATIVES
2936.28.0000	VITAMIN E (TOCOPHEROLS AND RELATED COMPOUNDS WITH VITAMIN E ACTIVITY) AND ITS DERIVATIVES
2936.29.1000	FOLIC ACID
2936.29.1510	NIACIN (PHARMACEUTICAL GRADE)
2936.29.1520	NIACIN (EXCLUDING PHARMACEUTICAL GRADE)
2936.29.1530	NIACINAMIDE
2936.29.2000	OTHER AROMATIC OR MODIFIED AROMATIC VITAMINS AND THEIR DERIVATIVES
2936.29.5020	VITAMINS D AND THEIR DERIVATIVES
2936.29.5050	OTHER VITAMINS (EXCLUDING AROMATIC AND MODIFIED AROMATIC) AND THEIR DERIVATIVES
2936.90.0110	PROVITAMINS
2936.90.0150	OTHER INCLUDING NATURAL CONCENTRATES
3004.50.5005	OTHER MEDICAMENTS CONTAINING VITAMINS OR OTHER PRODUCTS OF HEADING 2936 NESOI, FOR VETERINARY USE
3004.50.5010	SINGLE VITAMINS COMBINED WITH MINERALS OR OTHER NUTRIENTS
3004.50.5020	SINGLE VITAMINS, NOT COMBINED WITH MINERALS OR OTHER NUTRIENTS
3004.50.5030	MULTIPLE VITAMINS COMBINED WITH MINERALS OR OTHER NUTRIENTS
3004.50.5040	MULTIPLE VITAMINS NOT COMBINED WITH MINERALS OR OTHER NUTRIENTS

**TABLE 2:** Perceived risk of ingredients imported into the United States

Low^a^	Moderate^b^	High^c^
Amino acids (aromatic, Lys, Met)	Oilseed meals and flours	Choline
Grain co‐products (flaked, germ, gluten feed, malt)	Soy oil	Grain co‐products (groats, hulls, middlings)
Oil cakes (corn, cottonseed, rapeseed, sunflower)	Soy oilcake	Oilseed co‐products (hulls)
Oils (flaxseed, linseed, safflower, sunflower)		Whole and ground grains
Vitamins (A, B1, B2, B3, B3 and B5, B6, B9, C, D, E, aromatic, other)		Whole oilseeds

^a^ Processed ingredients which undergo either thermal or chemical processing during the manufacturing process.

^b^ Processed ingredients which undergo thermal or chemical processing but are able to maintain virus for an extended period of time.

^c^ Unprocessed ingredients.

### 2.2 Current regulations for non‐animal origin feed and ingredients imported from ASFV‐positive regions

To understand the complex environment of importation requirements across three countries, the guidelines from the United States, Canada and Australia were initially reviewed. Canada and Australia were selected for comparison because, similar to the United States, they are countries free from ASFV but depend on imports from ASFV‐positive countries. They also had implemented significant policies to prevent disease entry through ingredients. Independent interviews were also conducted with representatives in each country representing the federal government, feed industry and swine industry. Positions interviewed to compile this information from the United States include USDA APHIS‐PPQ National Policy Manager, USDA APHIS‐VS Director of Animal Product Imports, American Feed Industry Association Vice President of Public Policy and Education, and National Grain and Feed Association Senior Vice President of Feed Services. Positions interviewed from Canada include Canadian Food Inspection Agency Programs and Policy Branch National Manager, Canadian Food Inspection Agency Animal Health Risk Assessment and Intelligence Section Risk Analyst, Canadian Food Inspection Agency Foreign Animal Disease Section National Manager, Canadian Pork Council, Animal Nutrition Association of Canada Technical Services Director and Animal Nutrition Association of Canada Executive Director. Positions interviewed from Australia include Australian Department of Agriculture, Water, and the Environment Data Analyst, Australia Department of Agriculture Program Lead for Biosecurity and Social Science, SunPork Australia Veterinarian and African Swine Fever Liaison to Australian Pork Limited, SunPork Australia Nutritionist, Australian Pork Limited Manager of Production Stewardship and Stock Feed Manufacturers’ Council of Australia Executive Officer.

## 3 RESULTS AND DISCUSSION

### 3.1 The volume and path of non‐animal origin feed and ingredients imported from countries with ASFV

**FIGURE 1:**
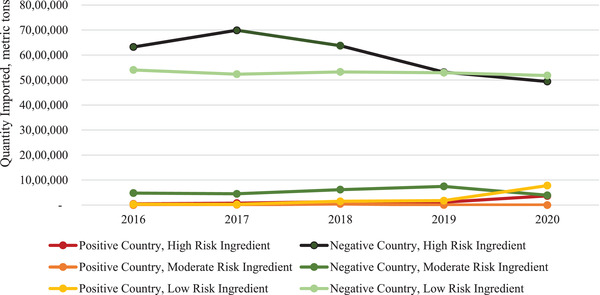
Ingredients imported from ASFV‐free and positive countries from 2016 to 2020.^a,b^ ^a^Quantities (metric tons) imported were obtained from the US International Trade and Tariff Database. ^b^African swine fever status was determined based on presence of cases in a country during each year as reported by the OIE‐WAHIS Quantitative Data database

**FIGURE 2:**
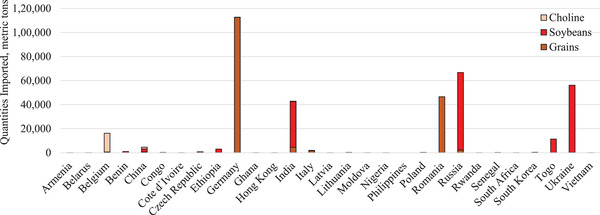
High‐risk ingredients imported from ASFV‐positive countries in 2020.^a,b^ ^a^Quantities (metric tons) imported were obtained from the US International Trade and Tariff Database. ^b^African swine fever status was determined based on presence of cases in a country during each year as reported by the OIE‐WAHIS Quantitative Data database

**FIGURE 3:**
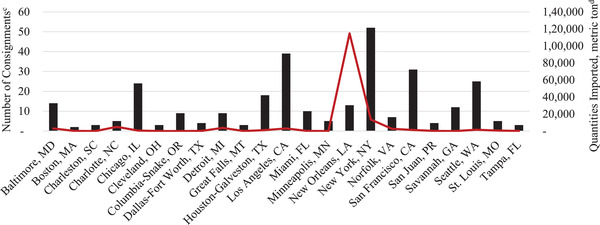
Port of entry high‐risk ingredients imported into the United States from ASFV‐positive countries in 2020.^a,b^ ^a^Quantities (metric tons) imported were obtained from the US International Trade and Tariff Database. ^b^African swine fever status was determined based on presence of cases in a country during each year as reported by the OIE‐WAHIS Quantitative Data database. ^c^Number of consignments are represented by the black bars. ^d^Quantities imported are represented by the red line

From 2016 to 2020, the quantity of feed ingredients imported into the United States ranged from 11.6 to 12.8 million metric tons per year (Figure 1). This quantity does not differentiate between those ingredients intended for human consumption, swine diets or other livestock and pet food diets. During the same time frame, the total quantity imported from ASFV‐positive countries ranged from 0.8 to 1.1 million metric tons per year. The greatest quantity imported from ASFV‐positive countries was in 2020, with the increase from recent years due to the growing number of United States’ trade partners that were impacted by the disease. In 2020, the United States imported ingredients that could be used in swine feed from 29 of the 50 countries that were ASFV‐positive, some of which are critical to US trade (Figure 2). The greatest quantity of high‐risk imported ingredients came from Germany, with all the incoming product consisting of grains. Relatively significant quantities of soybeans were imported from Russia, Ukraine and India in 2020, as well as grains from Romania. Choline was largely sourced from Belgium. Notably, fewer than 5000 metric tons of high‐risk ingredients were imported from China in 2020. These high‐risk ingredients were imported into 23 different US ports (Figure 3). The Port at New Orleans imported the greatest quantity of high‐risk ingredients from ASFV‐positive countries at more than 114,000 metric tons of product. These shipments contained 9 metric tons of barley imported from Italy and likely destined for human food and 114,775 metric tons of certified organic soybeans from Ethiopia, Russia, and Ukraine (Table 3). The source of these ingredients (i.e. region of a specific country) and transportation methods could not be further determined prior to their Port of Loading. Interestingly, the Ports of Loading for these shipments were from India and Turkey. This demonstrates that even when a country of origin can be determined for an ingredient, its growing conditions, transportation and other factors relating to the likelihood of ASFV contamination is unlikely to be known. These three shipments arrived at the Port of New Orleans in jumbo bags (45) or bulk (1) consignments after at least 32.4 days at sea. Part or all of each shipment was purchased by a single company, Sunrise Foods International, an entity based in Canada. These soybeans would likely require crushing prior to use in swine diets because whole, unprocessed soybeans have poorly available nutrients to pigs (Liener, 1994). Therefore, it is unlikely that any of the high‐risk ingredients received into the Port of New Orleans in 2020, which represents 31.4% of all high‐risk ingredients imported, was consumed by pigs. Moreover, it is improbable that the remaining high‐risk ingredients imported into other ports in 2020 were consumed by pigs due to the small volume of each consignment. For example, the Port of New York had the greatest number of consignments, or individual shipments of high‐risk ingredients. However, these consignments totalled to fewer than 14,000 metric tons of product, with most of the product arriving in small quantities that would be unlikely to be destined for animal feed.

**TABLE 3:** Unprocessed ingredients from ASFV‐positive countries imported into the Port of New Orleans in 2020^a^

Ingredient	Country of origin	Port(s) of loading	Days at sea	Consignments	Form	Volume, metric ton	Importer	Buyer
Barley	Italy	Sines, Portugal	22.2	29	Boxes	9	Italfoods, Inc	Frantoio Oleario Bartolini Emilio
Certified organic soybeans	Ethiopia	Jawaharal Nehru, India	45.1	1	Jumbo bags	220	Sunrise Foods International	Sunrise Foods International
	Russia	Jawaharal Nehru, Valencia, and Mundra, India	45.0	29	Jumbo bags	63,462	Leche Gloria Sociedad Anonima Glorai S A, Sunrise Foods International	Leche Gloria Sociedad Anonima Glorai S A and Norman Krieger, Inc., Sunrise Foods International
	Ukraine	Samsun, Turkey	32.4	16	Jumbo bags and bulk	51,093	Sunrise Foods International	Sunrise Foods International

^a^ Importer and buyer information was obtained from the US Import database.

**FIGURE 4:**
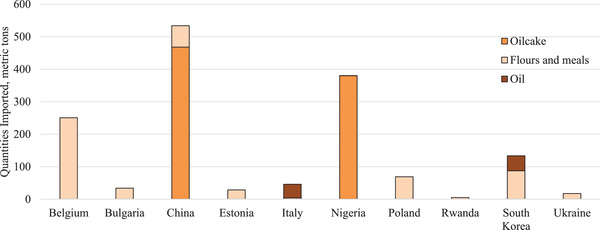
Moderate‐risk ingredients imported from ASFV‐positive countries in 2020.^a,b^ ^a^Quantities (metric tons) imported were obtained from the US International Trade and Tariff Database. ^b^African swine fever status was determined based on presence of cases in a country during each year as reported by the OIE‐WAHIS Quantitative Data database

The moderate‐risk ingredients were imported from 10 different ASFV‐positive countries (Figure 4). Soy oilcake was imported from China and Nigeria; soy flour and meals were imported from Belgium, Bulgaria, China, Estonia, Poland, Rwanda, South Korea and Ukraine and soy oil was imported from Italy and South Korea. The combined quantity of moderate‐risk ingredients from ASFV‐positive countries totalled less than 1500 metric tons. While a risk exists that these non‐animal origin ingredients may be contaminated with ASFV, these data support the USDA's assertion that there is a negligible risk for ingredients to serve as a fomite for ASFV entry into naïve pigs in the United States (USDA‐APHIS‐VS, 2019).

### 3.2 Current regulations for non‐animal origin feed and ingredients imported from ASFV‐positive regions

#### 3.2.1 United States of America

Non‐animal origin feed ingredients imported into the United States falls under two government jurisdictions. The FDA is responsible for the importation of non‐animal‐origin ingredient and complete feeds. Alternatively, the USDA Animal & Plant Health Inspection Service (APHIS) includes foreign animal disease prevention and surveillance and partners with US Customs and Border Protection to enforce importation requirements. Two agencies within USDA‐APHIS share this responsibility. Veterinary Services (VS) ‘regulates the importation of animals and animal‐derived materials to ensure that exotic animal and poultry diseases are not introduced into the United States’ (USDA APHIS‐VS, 2021). Currently, USDA APHIS‐VS prohibits most animal‐derived ingredients from being imported into the United States. In the rare instances where animal‐derived ingredients are allowed, such as vitamins coated in porcine‐derived gelatine to improve stability, special conditions or permits are issued by USDA APHIS‐VS to ensure the product has been processed completely to minimize the risk of disease. Meanwhile, another division of USDA APHIS, Plant Protection and Quarantine (PPQ) ‘regulates the importation of plants and plant products…from the risks associated with the entry, establishment, or spread of animal and plant pests or noxious weeds’ (USDA APHIS‐PPQ, 2021). The USDA APHIS‐PPQ specifies in its *Seeds not for Planting* guide that seeds from certain countries to be accompanied by phytosanitary certificates to eliminate the risk of pests, such as Khapra beetles in soybeans from Nigeria (USDA APHIS, 2020). When necessary, products may need to undergo more strenuous processes. In their *Miscellaneous and Processed Products Import Manual*¸ APHIS‐PPQ specifies that peanuts from Argentina or Brazil must be commercially blanched at 84˚C for 12 min to alleviate concerns of peanut stripe virus (USDA APHIS, 2017). These manuals do not currently have requirements that address animal pathogens in plant‐based products.

#### 3.2.2 Canada

Canada developed their risk management strategy based on the concept of strengthening the country's protection against ASFV entry in ingredients, but not eliminating the risk altogether. Their import policy was implemented after an extensive literature review and importer surveys to better understand the number of importers affected and their understanding of pathogen transmission in feed. Canada requires an import permit for any raw or unprocessed grains and oilseeds and any plant‐based meal of concern imported from an ASFV‐positive country. They also recognize regionalization with 12 countries. Regionalization is permitted in countries who participate in the European Union's Commission Implementing Regulation 2021/605 and 2021/1205, which designates restricted zones within a country based on the disease status of the wild and domestic swine populations. This permit must be accompanied by a completed questionnaire demonstrating certain conditions have been met either in the country of origin and certified by a competent authority (a representative of a governing body guaranteeing requirements have been met) or upon arrival at a Canadian port. The required conditions include (1) heat treatment with a minimum temperature of 70°C for 30 min or 85°C for 5 min or holding of the product should occur for a minimum of 20 days at 20°C or 100 days at 10°C and (2) prevention of cross‐contamination via unidirectional traffic at the processing facility, single use containers or bulk containers with plastic liners and no additions of unprocessed ingredients added to the finished product.

**FIGURE 5:**
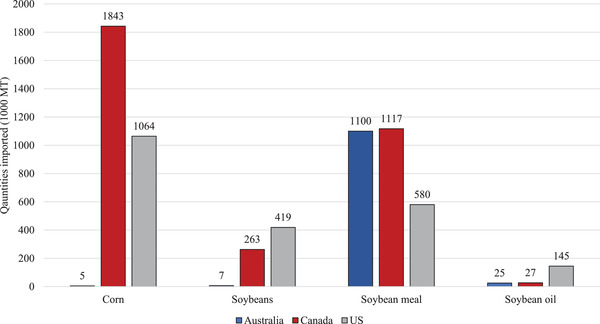
Bulk commodities imported into Australia, Canada and the United States in 2019/2020.^a^ ^a^Quantities imported were obtained from the USDA Foreign Agricultural Service – Production, Supply and Distribution database

Canada did not introduce a new regulation to implement their import policy. Under their *Health of Animals Act* and *Health of Animals Regulations*, the Minister of Agriculture can declare Secondary Control Zones (SCZ) to prevent disease entry into Canada. By declaring certain zones or countries areas of concern regarding ASFV, the Canadian Food Inspection Agency can create restrictions when importing certain ingredients from ASFV‐positive countries. The amendments to *Health of Animals Act* and *Health of Animals Regulations* allowed for rapid implementation of the new policies; however, more than just the swine industry would be affected by the new import requirements. The swine and feed industry met with the National Farm Animal Care Council (NFACC), a unified organization representing all livestock sectors, to discuss their need for import policies from ASFV‐positive countries. There was no opposition from any other industries which also eased the way for the Minister of Agriculture to approve the new policies. Canada only has 6 marine ports compared to 50 within the United States Canada, like the United States also imports a relatively low amount of bulk grains and oilseeds (Figure 5).

Those interviewed could not estimate a cost associated with the import policy. Any costs associated with increased holding time at port or additional processing are incurred by the importer. Increased costs at the port would trickle down to the buyers of the finished product; however, no analysis has been conducted to better understand the economic difference since this program has been implemented. All Canadian representatives stated that they did not believe a policy similar to Canada's would be practical to implement in the United States due to the greater complexity of grain trade and marine ports that exist in the United States.

#### 3.2.3 Australia

Australia implemented import control measures approximately 20 years ago to reduce the risk of introducing foot and mouth disease virus, bovine spongiform encephalopathy and plant pests, such as the Khapra beetle. After ASFV was identified in Southeast Asia, the Australian Department of Agriculture reviewed their policies and confirmed their existing rules would effectively prevent ASFV entry in ingredients so no new policies were needed. Current policies include that all imported cereal grains and oilseeds must be either processed at the port of entry or have an import permit from the country of origin signed by a competent authority that the product was effectively processed. All foreign facilities that process ingredients bound for Australia must be audited by the Australian government every 2 years at the cost to the importing facility (∼$10,000 AUD/audit). Accepted processing methods include (1) heating the product to a core temperature of 85°C for 10 min if it originates from a low‐risk country or to 100°C for 30 min if it originates from a high‐risk country; (2) pH reduction by 3.3 or (3) irradiation of 50 kGy. Australian pork industry officials interviewed described that even with these import restrictions in place, they frequently implemented their own holding time mitigation strategies due to their lack of confidence in effective processing by importing countries. The Australian government is currently conducting a cost:benefit analysis to determine the value of mitigation strategies to prevent ASFV. However, their analysis does not consider the role of feed or ingredients as a vector of ASFV entry or transmission. All Australian representatives stated that they did not believe a policy similar to Australia's would be practical to implement in the United States due to the size of the US feed and pork industries. In fact, both regulators and industry representatives alike expressed their concern that existing policies reduced the ability for pork producers to have access to low‐cost ingredients, which limited the industry's domestic growth and international competitiveness.

In comparison to Canada and Australia, the US regulations may appear lacking. However, there is a growing proportion of production companies taking innovative steps to secure their ingredient supply chains. In a preliminary survey, which represented companies owning one‐third of the pigs marketed annually, over half of the participants utilized holding times for imported vitamins, minerals and supplements from ASFV‐positive countries. Moreover, half of the participants were working towards sourcing only domestic grains and oilseeds. Other companies have privately been tracing their ingredient supply chain back to the original sources as well as quarantining those ingredients in segregated warehouses. Ingredient tracing programs allow the entire supply chain, including the veterinarians and producers to have more confidence in the quality and disease status of imported ingredients (Patterson et al., 2019). Furthermore, the swine industry has continued to support proactive research to lessen any gaps of knowledge. Blomme et al. (2022) looked specifically at soy ingredients imported into the United States from foreign animal disease positive countries beyond the ASFV positive countries mentioned in this study. While Schambow et al. (2022) developed a model to predict ASFV entry more accurately through non‐animal origin feed ingredients. Innovative steps such as these can take the place of government regulations and can instead demonstrate the unity and forward thinking of the swine industry.

## 4 LIMITATIONS

As with any study, there are limitations which must be addressed. Due to time restraints, a limited number of individuals were able to be interviewed. As the objectives were primarily focused on regulatory structure in certain countries, the interviews primarily focused on governing regulatory bodies and national organizations. This limited representation from individual swine producers and veterinarians, which may view the challenges facing the swine industry differently than those interviewed.

## 5 CONCLUSION

There continues to be a small, but realistic threat for African swine fever virus entry into the United States through non‐animal origin feed ingredients. In 2020, moderate‐ and high‐risk ingredients from ASFV‐positive countries represented only 3.1% of all ingredients imported into the United States. While the United States has policies to prevent foreign entry of plant pests and diseases, Canada and Australia have been more aggressive in their regulatory approach to prevent animal pathogens in plant‐based ingredients. Industry led practices can be developed to disallow use of high‐ and medium‐risk ingredients from ASFV‐positive countries in swine feeds and require the use of mitigation strategies in low‐risk ingredients if there is a high demand from producers and industry leaders.

## CONFLICT OF INTEREST

The authors declare no conflicts of interest in the completion and reporting of results for this investigation.

## Data Availability

The data that support the findings of this study are available from corresponding author upon reasonable request.

## References

[tbed14473-bib-0001] Blomme, A. K. , Jones, C. K. , Gebhardt, J. T. , Woodworth, J. C. , & Paulk, C. B. (2022) Assessment of soy‐based imports into the United States and associated foreign animal disease status. Transboundary and Emerging Diseases, 69, 137–148. 10.1111/tbed.14284 34369092 PMC9290452

[tbed14473-bib-0002] Dee, S. A. , Bauermann, F. V. , Niederwerder, M. C. , Singrey, A. , Clement, T. , de Lima, M. , Long, C. , Patterson, G. , Sheahan, M. A. , Stoian, A. M. M. , Petrovan, V. , Jones, C. K. , De Jong, J. , Ji, J. , Spronk, G. D. , Minion, L. , Christopher‐Hennings, J. , Zimmerman, J. J. , …, & Diel, D. G. (2018). Survival of viral pathogens in animal feed ingredients under transboundary shipping models. PLoS One, 13(3), e0194509. 10.1371/journal.pone.0194509 29558524 PMC5860775

[tbed14473-bib-0003] Dee, S. , Shah, A. , Jones, C. , Singrey, A. , Hanson, D. , Edler, R. , Spronk, G. , Niederwerder, M., & Nelson, E. (2021) Evidence of viral survival in representative volumes of feed and feed ingredients during long‐distance commercial transport across the continental United States. Transboundary and Emerging Diseases, 69, 149–156. 10.1111/tbed.14057 33763985 PMC9290857

[tbed14473-bib-0004] Dee, S. A. , Clement, T. , Schelkopf, A. , Nerem, J. , Knudsen, D. , Christopher‐Hennings, J. , & Nelson, E. (2014). An evaluation of contaminated complete feed as a vehicle for porcine epidemic diarrhea virus infection of naïve pigs following consumption via natural feeding behavior: Proof of concept. BMC Veterinary Research, 10, 176. 10.1186/s12917-014-0176-9 25091641 PMC4363994

[tbed14473-bib-0005] EFSA. European Food Safety Authority . (2021). Ability of different matrices to transmit African swine fever virus. https://efsa.onlinelibrary.wiley.com/doi/epdf/10.2903/j.efsa.2021.6558. Accessed 15 July 2021. 10.2903/j.efsa.2021.6558 PMC8077412 33936310

[tbed14473-bib-0006] Jones, C. K. , Stewart, S. C. , Woodworth, J. C. , Dritz, S. S. , & Paulk, C. B. (2019) Validation of sampling methods in bulk feed ingredients for detection of swine viruses. Transboundary and Emerging Diseases, 67, 1–5. 10.1111/tbed.13326 31403747 PMC7003878

[tbed14473-bib-0007] Liener, I. E. (1994) Implications of antinutritional components in soybean foods. Critical Reviews in Food Science and Nutrition, 34, 31–67. 10.1080/10408399409527649 8142044

[tbed14473-bib-0008] Niederwerder, M. C. , Stoian, A. M. M. , Rowland, R. R. R. , Dritz, S. S. , Petrovan, V. , Constance, L. A. , Gebhardt, J. T. , M. Olcha , Jones, C. K. , Woodworth, J. C. , Fang, Y. , Liang, J. , & Hefley, T. J. (2019). Infectious dose of African swine fever virus when consumed naturally in liquid or feed. Emerging Infectious Diseases, 25, 891–897. 10.3201/eid2505.181495 30761988 PMC6478231

[tbed14473-bib-0009] Patterson, G. , Niderwerder, M. C. , & Dee, S. A. (2019). Risks to animal health associated with imported feed ingredients. Journal of the American Veterinary Medical Association, 254, 790–791. 10.2460/javma.254.7.790 30888283

[tbed14473-bib-0010] Schambow, R. A. , Samperdro, F. , Urriola, P. E. , van de Ligt, J. L. G. , Perez, A. , & Shurson, G. C. (2022). Rethinking the uncertainty of African swine fever virus contamination in feed ingredients and risk of introduction into the United States. Transboundary and Emerging Diseases, 69, 157–175. 10.1111/tbed.14358 34689419

[tbed14473-bib-0011] Soy Transportation Coalition . Classes of vessels and cargo capacity. https://www.soytransportation.org/Stats/Ocean_VesselClasses.s. Accessed 15 July 2021.

[tbed14473-bib-0012] Stoian, A. M. M. , Zimmerman, J. , Ji, J. , Hefley, T. J. , Dee, S. A. , Diel, D. G. , Rowland, R. R. R. , & Niederwerder, M. C. (2019) Half‐life of African swine fever virus in shipped feed. Emerging Infectious Diseases, 25, 2261–2263. 10.3201/eid2512.191002 31524583 PMC6874236

[tbed14473-bib-0013] USAHA. United States Animal Health Association . (2020) Feed import restrictions to protect against African Swine fever importation in feed. https://www.usaha.org/upload/Resolution/2020/2020Resolution_3_FED_2_Feed_I.2.pdf Accessed 15 July 2021.

[tbed14473-bib-0014] USDA APHIS‐PPQ. United States Department of Agriculture Animal and Plant Health Inspection Service . Plant Import Information . (2021) https://www.aphis.usda.gov/aphis/ourfocus/planthealth/import‐information. Accessed 15 July 2021.

[tbed14473-bib-0015] USDA APHIS‐VS. United States Department of Agriculture Animal and Plant Health Inspection Service Veterinary Service . (2021). Import animal products. https://www.aphis.usda.gov/aphis/ourfocus/animalhealth/import‐animals‐products/ct_import_animal_products. Accessed 15 July 2021.

[tbed14473-bib-0016] USDA‐APHIS‐VS. United States Department of Agriculture Animal and Plant Health Inspection Service Veterinary Services . (2019) Qualitative assessment of the likelihood of African swine fever virus entry to the United States: Entry assessment. https://www.aphis.usda.gov/animal_health/downloads/animal_diseases/swine/asf‐entry.pdf. Accessed 15 July 2021.

[tbed14473-bib-0017] USDA‐APHIS. United States Department of Agriculture Animal and Plant Health Inspection Service (2020) Seeds not for planting. https://www.aphis.usda.gov/import_export/plants/manuals/ports/downloads/seeds_not_for_planting.pdf Accessed 15 July 2021.

[tbed14473-bib-0018] USDA‐APHIS. United States Department of Agriculture Animal and Plant Health Inspection Service . (2017) Miscellaneous and processed products manual. https://www.aphis.usda.gov/import_export/plants/manuals/ports/downloads/miscellaneous.pdf. Accessed 15 July 2021.

[tbed14473-bib-0019] USDA‐APHIS. United States Department of Agriculture Animal and Plant Health Inspection Service . (2015) Swine enteric coronavirus introduction to the United States: Root cause investigation report. https://www.aphis.usda.gov/animal_health/animal_dis_spec/swine/downloads/secd_final_report.pdf. Accessed 15 July 2021.

